# Using an adaptive network-based fuzzy inference system for prediction of successful aging: a comparison with common machine learning algorithms

**DOI:** 10.1186/s12911-023-02335-9

**Published:** 2023-10-19

**Authors:** Azita Yazdani, Mostafa Shanbehzadeh, Hadi Kazemi-Arpanahi

**Affiliations:** 1 Health Human Resources Research Center, Shiraz University of Medical Sciences, Shiraz, Iran; 2 Clinical Education Research Center, Shiraz University of Medical Sciences, Shiraz, Iran; 3 Department of Health Information Management, School of Health Management and Information Sciences, Shiraz University of Medical Sciences, Shiraz, Iran; 4 Department of Health Information Technology, School of Paramedical, Ilam University of Medical Sciences, Ilam, Iran; 5 Department of Health Information Technology, Abadan University of Medical Sciences, Abadan, Iran

**Keywords:** Machine learning, Artificial intelligence, Neural networks, Fuzzy logic, Successful aging

## Abstract

**Introduction:**

The global society is currently facing a rise in the elderly population. The concept of successful aging (SA) appeared in the gerontological literature to overcome the challenges and problems of population aging. SA is a subjective and multidimensional concept with many ambiguities regarding its meaning or measuring. This study aimed to propose an intelligent predictive model to predict SA.

**Methods:**

In this retrospective study, the data of 784 elderly people were used to develop and validate machine learning (ML) methods. Data pre-processing was first performed. First, an adaptive neuro-fuzzy inference system (ANFIS) was proposed to predict SA. Then, the predictive performance of the proposed model was compared with three ML algorithms, including multilayer perceptron (MLP) neural network, support vector machine (SVM), and random forest (RF) based on accuracy, sensitivity, precision, and F-score metrics.

**Results:**

The findings indicated that the ANFIS model with gauss2mf built-in membership function (MF) outperformed the other models with accuracy, sensitivity, precision, and F-score of 91.57%, 95.18%, 92.31%, and 92.94%, respectively.

**Conclusions:**

The predictive performance of ANFIS is more efficient than the other ML models in SA prediction. The development of a decision support system (DSS) using our prediction model can provide healthcare administrators and policymakers with a reliable and responsive tool to improve elderly outcomes.

## Introduction

Old age is a natural stage of human life characterized by physiological, psychological, and social changes [1]. The World Health Organization (WHO) recognizes 60 years and older as elderly, although some developed countries consider the age of 65 as the elderly threshold [2, 3]. According to the WHO, the elderly population is increasing, accounting for approximately 16% of the global population by 2050 [4]. At present, dramatic socio-demographic and lifestyle changes are observed in the world’s population, especially in developed countries, and the world is facing a conversion from an aged to a super-aged population [5, 6]. Iran is not excluded from this change, and because of the alterations in epidemiologic characteristics of diseases, it is predicted to face an unexpected surge in the elderly population in the next two decades [7, 8]. As the WHO stated, if 7% of the population of a country is above 60 years of age, it is an old country [9]. According to the 2016 census in Iran, the number of people aged 60 years and older reached 7,414,091, accounting for 9.28% of the total population, and thus Iran is one of the oldest countries in the world [10].

The growth of the elderly population is due to scientific advances in medicine [11]. Aging is not a disease in itself, but if the health of the elderly population is neglected, it will have profound consequences on society’s health and economic conditions [12]. Negative outcomes of the elderly population growth include changes in various aspects of the elderly’s health such as reduced quality of life (QoL), dependence on others in performing daily tasks, increased mental disorders due to mourning multiple losses, job change, and increased stress, depression, and suicide [13–15]. With a rise in the elderly’s life expectancy, the probability of passing this period with disability and illness increases, and their need for hospital services are much higher than in other age groups [16]. Furthermore, the rise in the elderly population has increased the burden of non-communicable diseases in the world, especially in developed countries, thereby increasing the deaths due to these diseases [17]. The rapid growth of the elderly population has led to the transmission of epidemiology because the rapid increase in life expectancy has replaced heart diseases and cancer as the leading causes of death compared to other factors. As a result, although the risks of infectious diseases have decreased in these countries, the rate of chronic degenerative diseases has increased [18–20]. Therefore, it is crucial to deviate from the undesirable perception of the elderly period as one full of disease, disability, and loss of functions, and shift the attention to vigorous aging and improving the QoL of the elderly [21].

In this regard, successful aging (SA) is an effective strategy stressing healthy longevity and QoL in the elderly [22]. The concept of SA has been construed from various standpoints, and while its description has evolved in research over the past 50 years, there remains no consensus as to its definition [23]. Generally, a person with SA is one who has good physical, cognitive, and social functioning without major diseases which disrupt the life of this age group [24]. SA was introduced by Robert Havirast in 1963. He defined active aging as the achievement of maximum satisfaction in the later stages of life [25]. Raw and Kahn provided one of the most accepted theories about SA. They stated that SA is the end of a chain that extends from pathology to normal aging and from normal aging to SA [26]. As for separating the effects of disease from the aging process, Raw and Kahn declared that defects or diseases that occur naturally in old age must be separated from the pathological process [27]. Based on this model, SA is presented with three components: (1) being free from disability and diseases, (2) having a high level of action and mental activity, and (3) having social acceptance (both social activity and productive role) [28]. 

The emphasis of many studies on SA has shifted from a single dimension (disease existence or functional deterioration) to the multi-dimensional concept of SA, which is consistent with the WHO’s definition of health, whereby health is considered a state of complete physical, mental, social, and spiritual well-being [29]. However, due to the inherent ambiguity in the definition of this complex and multidimensional phenomenon, its definition has proved to be difficult task [30]. Creating a model for SA prediction can be effective in improving the predictive performance of healthcare providers, which can be very useful in timely diagnosis, focus, and adjustment of various factors causing this phenomenon in the elderly and, thus, increasing the QOL in this age group [31, 32]. Health experts make use of artificial intelligence (AI)-based solutions to support diagnosis and make recommendations in situations where clinical decision-making is full of uncertainty [33, 34]. In th is regard, as noted in [35, 36] integrating fuzzy logic and neural network is the greatest and most effective way of dealing with uncertainty. However, using these two techniques separately can have some weaknesses. Adaptive neuro-fuzzy inference system (ANFIS) is a hybrid ANN combined with fuzzy systems. ANFIS is a fuzzy inference system (FIS) implemented in the framework of adaptive networks to solve function approximation problems. It combines connectionist structure of neural network with human-like reasoning ability of fuzzy logic to deal with ambiguity and nonlinear relationships and works as a universal estimator [37, 38]. It depends on data that learn the rules and membership functions. This inference systems model for ANFIS is Takagi–Sugeno FIS, developed in the early 1990s [39]. The idea behind the ANFIS method is the need to provide a combined and effective solution by merging the learning of neural networks with the advantage of fuzzy if-rules that reflect human thought and knowledge. The learning networks in this model are based on mathematical computations capable of solving complex problems [40]. From the review of literature, it is observed that, ANFIS as a hybrid AI system widely applied in predicting the state and progression of different diseases and clinical conditions [41–46]. Results of these studies demonstrated that adopting ANFIS-based systems by medical experts may significantly decrease diagnostic errors. Such systems are more accurate than machine learning (ML) techniques. To our knowledge there has not been any study using ANFIS technique to predict SA. Therefore, the main purpose of this study was to develop an ANFIS-based model to predict SA and then compare its predictive performance with several well-known ML algorithms, including multilayer preception (MLP), support vector machine (SVM), and random forest (RF) to select the most accurate model for SA prediction.

This manuscript is structured as follows: First, the dataset is elaborated. The ML techniques used in this paper are then described in detail. After that, the results of comparing ML techniques are delineated. Finally, the most accurate model for SA prediction based on the results of performance evaluation metrics is presented.

## Methods

### Study design and setting

**Fig. 1:**
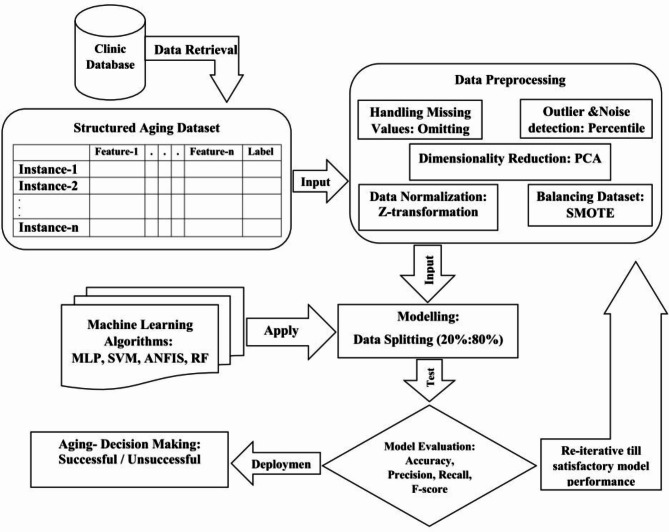
Research methodology flowchart

This was an applied and retrospective study conducted in 2022 using a dataset, including 1115 elderly people in a database from Abadan University of Medical Sciences, Abadan, Iran, from January 2016 to August 2021. This study aimed to assess the performance of several ML algorithms for SA prediction. The SA concept in our study was according to Rowe and Kahn’s theory, which mainly includes the following three dimensions: physiological, cognitive psychological, and social function. In this study, according to WHO, people aged 60 years or older were considered elderly. Therefore, individuals aged less than 60 years and incomplete case records with more than 70% missing data were excluded. All modeling steps were implemented in MATLAB and Google Colab. The primary population in our study included 293 SA and 822 non-SA individuals. After applying the inclusion criteria, 973 individuals remained. A total of 744 and 229 cases were associated with non-SA and SA, respectively. A conceptual schema of our methodology is depicted in Fig. 1. 

### Research variables

The included cases were defined based on 28 independent features in eight categories and one dependent feature classified as SA (coded 1) or non-SA (coded 0).

Numerous features are collected for the aged people in the electronic medical record (EMR) database. Thus, first, the data dictionary of the database was investigated to completely comprehend the meanings of the data and select suitable features. The candidate features affecting SA were selected based on consultations with geriatrician specialists and studying the relevant literature. Predictor and outcome features are described in Table 1.

**Table 1:** The Primary list of features utilized in recognizing SA

Variable type	Variable Group	Variable name
Input	Demographic	Age (years), sex, educational level, marital status
	Socio-Economic	Occupation, income level, family support, insurance situation
	Presence illness	Blood pressure, cerebrovascular accident (CVA), osteopathic, eye disorders, renal disorders, liver disorders, muscle disorders, diabetes, cancer, convalescences, and other diseases
	Functional domain	Ability to perform activities of daily li ving (ADLs), sports activities, exercise time, and type of exercise.
	Sexual health	Sexual health assessment
	Lifestyle domain	Tension management, social and interpersonal relationships, performing disease prevention activities, physical activity, and exercise, assessment of nutritional status, assessment of mal-nutritional status, and general description of lifestyle.
	QoL	Physical health (general health, pain assessment, fatigue, physical dysfunction, physical function), mental and social health (satisfaction, social function, mental dysfunction), total description of the QoL
	Life Satisfaction	Life satisfaction assessment
Output	SA	(SA = 1), (non-SA = 0)

The sociodemographic and health-related variables are easily understandable and can be collected based on adults’ information and their medical records (Table 1). However, some physical, behavioral, and psychosocial factors such as the ability to perform activities of ADLs, life satisfaction, QoL, healthy lifestyle, nutrition assessment, and stress management were measured as follows: 

#### Ability to perform ADLs

This variable was evaluated by the Barthel index, which has 10 queries to evaluate an adult’s ability to perform elementary ADLs, e.g., feeding, bathing, dressing, going to the toilet, and transferring and maintaining continency unassisted [47]. The Barthel index ranges from 0 to 100. Scores of 0–20 indicate severe dependence, 20–60 complete dependence, 61–90 moderate dependence, 91–99 partial dependence, and 100 complete independence [48]. In our study, an independent person was someone who had a score of 100 based on the Barthel index.

#### Life satisfaction

Diener’s Satisfaction with Life Scale was used to measure adult life satisfaction. It has five items to measure the mental component of well-being. Each item has seven choices ranging from 1 to 7 (*strongly disagree* to *strongly agree*). Bayani et al. confirmed the validity of this tool for the Iranian population [49]. In the present study, a person satisfied with life would obtain a score of > 20 on this scale.

#### QoL

A 36-Item Short-Form scale (SF-36) was constructed to survey QoL and health status. This instrument contains 36 items that evaluate eight health concepts, including physical functioning, social functioning, physical role functioning, emotional role functioning, mental health, evaluations of vitality, physical pain, and general health. In addition to these sections, SF36 also provides two general measures of physical health (total physical component score (PCS)) and mental and social health (total mental component score (MCS)). The respondents’ scores in each domain vary from 0 to 100, and a higher score means a better QoL [50]. The validity and reliability of this questionnaire in the Iranian population have been confirmed [51, 52]. In our study, we considered a score of 70 as the cut-off point for QoL.

#### Healthy lifestyle

A healthy lifestyle is a multidimensional concept involving individual health and hygiene, carrying out daily work on one’s own, sports, healthy nourishment, spiritual health, stress management, social and interpersonal relationships, and spiritual and religious activities [53]. To measure an adult’s healthy lifestyle, the healthy lifestyle questionnaire was administered. In this 46-item instrument, a score of 42 to 98 represents an unfavorable, 99 to 155 shows a medium, and 156 to 211 denotes a desirable lifestyle. The validity and reliability of this instrument have been confirmed among Iranian people [9]. In this study, a desirable lifestyle would indicate a healthy lifestyle.

#### Nutrition status

The Mini Nutritional Assessment Questionnaire was used to measure the adults’ healthy nourishment status. In this instrument, a score of 12 or above shows that the adult is well-fed and needs no more intervention. A score of 8 to 11 indicates that the adult is in danger of malnourishment. A score of ≤ 7 denotes that the adult is malnourished. In our study, we considered 12 as the cut-off point for this variable.

#### Stress management

Using the Stress Management Questionnaire, the adults’ capability to cope with problematic and worrying circumstances was determined. This questionnaire scores stress management as low (ranging from 0 to 30), moderate (ranging from 31 to 39), and high (ranging from 40 to 50) classes. In this study, we considered 31 as the cut-off point for this variable.

#### Outcome variable

The dependent variable was SA (yes / no) as defined by Rowe and Kahn’s theory which has three main components [54]. Using this theory, adults meeting the following criteria were included: 1) lack of illness and infirmity (the criteria are met when the elderly have no disability and the number of chronic diseases is ≤ 2), 2), maintenance of high mental and physical function (in this domain, the adults had a Mini-Mental State Examination for Dementia Screening (MMSE-DS) score of normal and a Bartle index of > 90), and 3) continued engagement with life (this domain was determined based on employment, participation in social activities, religious activities, volunteering activities, and lifelong learning. The participants had to have at least three out of these five criteria) [55–59].

### Dataset preprocessing

Data preprocessing was performed in three steps as follows:

#### Handling missing values and outliers

Before using the data for modeling, data preprocessing, including outlier detection, was performed using the percentile method. In this way, out-of-range data were identified. Numbers “10” and “90” were considered thresholds. Finally, the value of 10% was chosen empirically by trial and error. The feature range was calculated, and the data at the top and bottom of 10% were deleted. Our dataset had three records with more than 70% missing. The missing records were deleted using an eliminated data objects method to handle missing values. 

#### Feature extraction 

The principal component analysis (PCA) feature extraction method was used. This method extracts important features (in the form of components) from a large set of features in a data set. The PCA actually extracts a low-dimensional set of features from a high-dimensional set to help capture more information with fewer variables. In this way, data visualization also becomes more meaningful. PCA is used in exploratory data analysis and to build predictive models. It is commonly used to reduce dimensionality by representing each data point on multiple principal components to obtain lower dimensional data while maintaining maximum data diversity. PCA is a common feature extraction method in data science. Technically, PCA finds the eigenvectors of a covariance matrix with the highest eigenvalues and then uses them to represent the data in a new subspace of equal or lesser dimensions. PCA transforms a matrix of n features into a new data set with fewer than n features. That is, it reduces the number of features by creating new and smaller variables that capture a significant part of the information contained in the main features [60].

#### Balancing the dataset

ML algorithms tend to produce inappropriate classifiers when faced with imbalanced datasets. We used the synthetic minority over-sampling technique (SMOTE) to deal with unbalanced data in the dataset. The SMOTE algorithm is the most popular technique to increase the size of the minority class by generating synthetic instances. This algorithm produces new instances for the minority class in the neighborhood of the existing instances in this class [61]. Finally, the ratio of classes in the dataset was equal.

### Developing prediction models

For SA prediction, four models of ANFIS, SVM, RF, and MLP were trained. These data-driven modeling techniques all benefit from advances in computer science. They are widely used in many fields such as biology, economics, and medicine [62]. We present a brief description of these four algorithms here.

#### ANFIS

**Fig. 2:**
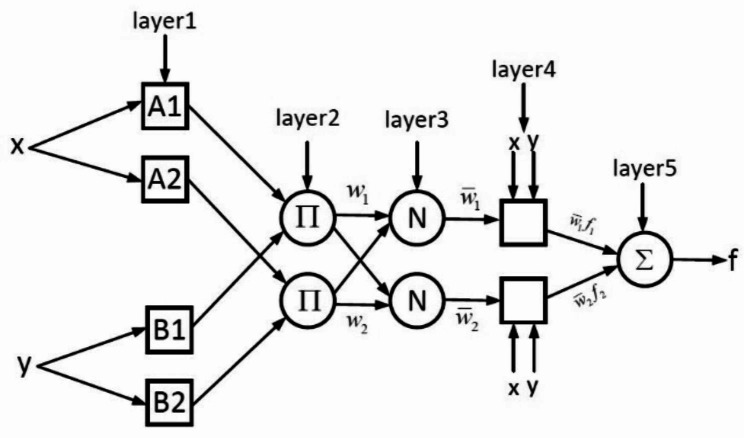
The equivalent structure of ANFIS ANFIS model, where *x* and *y* are inputs; A1, A2, B1 and B2 are fuzzy subsets; Π is the fixed nodes of Layer 2; Wi is the weight of a given fuzzy rule, fi; *N* is the fixed nodes of Layer 3; wi is the normalized weight; fi is the fuzzy rule; and f is the final output of the ANFIS model

We used the fuzzy C-means (FCM)-based ANFIS as a method that uses the advantages of neural network and fuzzy method approaches for modeling SA. This was done to show the high capability of this method in modeling medical problems. The ANFIS structure consists of five layers. The first layer performs the fuzzification. Fuzzification is the flow of assigning the numerical input of a system to fuzzy sets with some degree of membership. In this layer, the type and number of membership functions are specified, and all the existing fuzzy rules are produced. In the second layer, which can also be called the inference layer, the effect of each rule is calculated. The rules are defined in this layer. The effect of each rule is normalized according to the effect of other rules. In the fourth layer, the output of each rule is obtained, which calculates the weighted outputs. In the fifth layer, the outputs of the fourth layer are added to each other to form the output of the fuzzy system (Fig. 2). The fuzzy inference system is used to derive a set of rules that model the behavior of data, which uses FCM clustering to determine the number of rules and membership functions. ANFIS is an adaptable and trainable network that is quite similar in function to the fuzzy inference system. For simplicity, we assume that our fuzzy system has two inputs x and y and its output is f. Now, if the rules are as follows:

$$\begin{array}{l}Rule1:if\,x\,is\,{A_1}\,and\,y\,is\,{{\rm{B}}_1}\,then\,{f_1} = {p_1}x + {q_1}y + {r_1}\\Rule2:if\,x\,is\,{A_2}\,and\,y\,is\,{{\rm{B}}_2}\,then\,{f_2} = {p_2}x + {q_2}y + {r_2}\end{array}$$

and if we use the centroid (centroid-of-gravity) method for defuzzification, the output will be as follows:

1 $$\begin{array}{*{20}{c}} {f=\frac{{{w_1}{f_1}+{w_2}{f_2}}}{{{w_1}+{w_2}}}=\bar {w}{f_1}+{{\bar {w}}_2}{f_2}}&{st}&{{{\bar {w}}_1}=\frac{{{w_1}}}{{{w_1}+{w_2}}},{{\bar {w}}_2}=\frac{{{w_2}}}{{{w_1}+{w_2}}}} \end{array}$$

Then, the equivalent structure of ANFIS will be as follows (see Fig. 2):

The output of the layers is as follows:

**Layer 1**: Here, the inputs are membership functions. Each suitable parametric function can be selected; in most cases, the Gaussian fuzzy membership function is used.

**Layer 2**: The output of this layer is the multiplication of the input signals, which is equivalent to the “if” part of the set of rules. The second and third layers are for inference between IF and Then. The second layer performs the product П operation on the membership degrees to calculate the ring strength () of each rule.

**Layer 3**: This layer normalizes the output of the previous layer.

**Layer 4**: Calculation of the following equation.

2 $${\bar {w}_i}{f_i}={\bar {w}_i}\left( {{p_i}x+{q_i}y+{r_i}} \right)$$

**Layer 5**: This layer is the overall output of rules calculated in the previous layer.

ANFIS learns by updating tunable parameters which are MF parameters and consequent parameters (p; q; r). The nodes of Layers 1 and 4 are trainable, whereas the nodes of the rest of the layers are fixed.

The network can be trained in different ways. There are two methods, gradient descent and hybrid, In MATLAB. In this research, the hybrid method was adopted. The combined method is a combination of gradient descent and minimum mean square error (MMSE) methods, which has higher speed and accuracy. ANFIS receives the extracted components from the PCA technique as the input; 80% of the number of instances was considered as the training set, and 20% as the test set. The training and test sets were randomly selected. The performance of ANFIS involves the selection of the number and shape of MFs. These are the most influential on the computational complexity and accuracy of the developed ANFIS-based model [63]. Hence, we examine the role of the eight shapes of MFs on the performance of ANFIS in solving the aging classification problem. And finally, we choose the MF that increases the modeling accuracy.

#### MLP

**Fig. 3:**
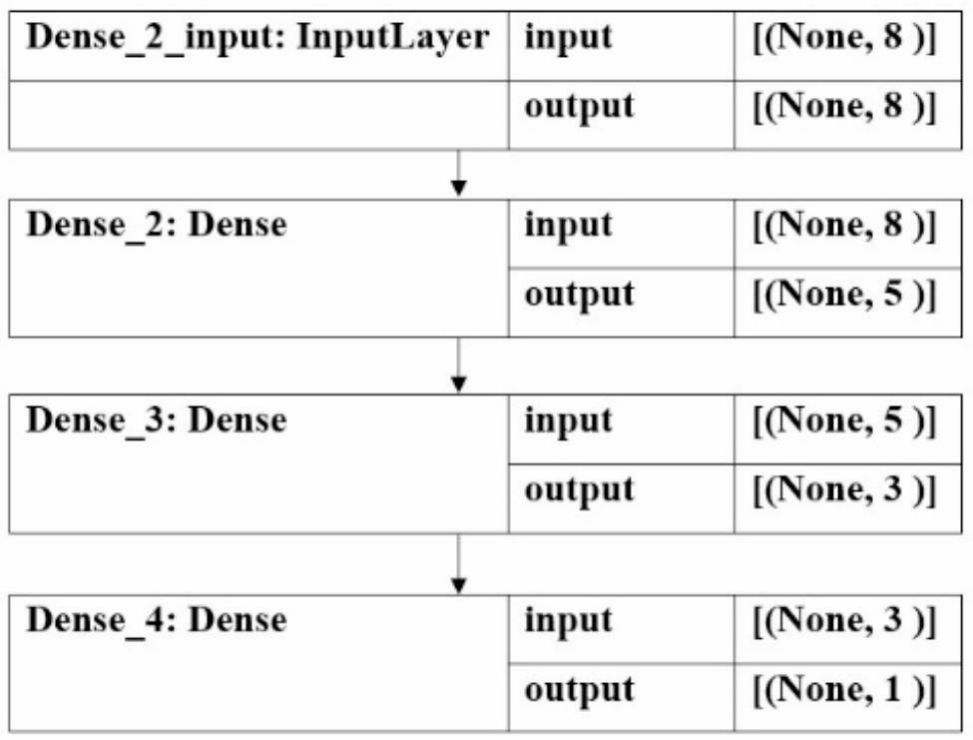
The proposed MLP architecture

The artificial neural network (ANN) is a major ML technique that plays a crucial role in performing nonlinear analysis in the diagnosis and prognosis of diseases and health conditions. MLP is a fully connected class of feedforward ANN. It consists of at least three layers of nodes: an input layer, a hidden layer, and an output layer. Except for the input nodes, each node is a neuron that uses a nonlinear activation function. MLP utilizes a supervised learning technique called backpropagation for training [64–67]. MLP was trained by 80% of the total data as a training dataset, which has been sorted randomly by the model. In this study, the network had two hidden layers and the activation function of the hidden layers was tanh; in the last layer, we used the sigmoid function. The Adam optimization algorithm was utilized in this network and the training rate was equal to ${10^{ - 4}}$. The training of the weights was performed through the stochastic gradient descent (SGD) algorithm, and the number of epochs was 25. A batch size of 32 was also selected. During the training, we considered 20% of the data validation data to avoid overtraining. The average training time per epoch was obtained as about four seconds. The MLP model is illustrated in Fig. 3.

#### SVM

SVM is a discriminative algorithm for categorizing both linear and non-linear data by first mapping each data point into n-dimensional features and then identifying the hyperplane separating the data points into different classes while maximizing the marginal intervals and minimizing classification errors [68]. The C and Kernel hyperparameters were used. The C values used were 1, 2, and 3, and the Kernels used were Poly Kernel, Normalized Poly Kernel, and Pearson VII function (PUK).

#### RF

This classifier is a bagging algorithm. RF is a meta-estimator that fits several decision tree classifiers under different samples of the dataset and uses the average to improve prediction accuracy and control over-fitting; as a result, the RF algorithm has a strong generalization. RF is suitable for processing large and high-dimensional data. The samples are chosen arbitrarily. In this algorithm, the subsample size is always equal to the original input sample size. Bootstrapping ensures that any decision tree inside the RF is diverse, dropping the RF variance [69, 70]. We set the maximum depth to zero, which means unlimited depth, and the number of iterations to 100, which is the number of trees in the forest. We tuned the bag size of the RF algorithm and performed three trainings. The bag size hyperparameter of RF was tuned to have values of 100, 75, and 50.

### Evaluation metrics

We employed the selected features to train the classifiers, and then evaluated their performance in terms of sensitivity, precision, F-measure, and accuracy (Table 2).

**Table 2:** Confusion matrix and definitions of terms used for measuring the models’ performance

		Predicted class	
		SA	non-SA
**Real class**	SA	**True Positive (TP)**: The number of cases with SA that are properly categorized as SA cases.	**False Positive (FP)**: The number of cases with non-SA that are wrongly categorized as SA cases.
	non-SA	**False Negative (FN)**: The number of cases with SA that are wrongly categorized as non-SA cases.	**True Negative (TN)**: The number of cases with non-SA that are properly categorized as non-SA cases.

These metrics are calculated based on Eqs. 3–6.

3 $$Sensitivity = {\rm{TPR}} = \frac{{{\rm{TP}}}}{{{\rm{TP}} + {\rm{FN}}}}$$

4 $$\text{A}\text{c}\text{c}\text{u}\text{r}\text{a}\text{c}\text{y}=\text{F}\text{P}\text{R}=\frac{\text{T}\text{P}+\text{T}\text{N}}{\text{T}\text{P}+\text{F}\text{P}+\text{T}\text{N}+\text{F}\text{N}}$$

5 $$\text{P}\text{r}\text{e}\text{c}\text{i}\text{s}\text{i}\text{o}\text{n}=\frac{\text{T}\text{P}}{\text{T}\text{P}+\text{F}\text{P}}$$

6 $$\text{F}-\text{M}\text{e}\text{a}\text{s}\text{u}\text{r}\text{e}=\frac{2\text{*}\text{P}\text{r}\text{e}\text{c}\text{i}\text{s}\text{i}\text{o}\text{n}\text{*}\text{S}\text{e}\text{n}\text{s}\text{i}\text{t}\text{i}\text{v}\text{i}\text{t}\text{y}}{\text{P}\text{r}\text{e}\text{c}\text{i}\text{s}\text{i}\text{o}\text{n}+\text{S}\text{e}\text{n}\text{s}\text{i}\text{t}\text{i}\text{v}\text{i}\text{t}\text{y} }$$

## Results

After the preprocessing process, the number of samples was reduced to 784 cases. In the final dataset, 392 cases were categorized into class 1 and 392 cases into class 0. According to the PCA method, eight components were selected to classify the data (Table 3). The explained variance is a statistical measure of how much variation in a dataset can be attributed to each of the principal components generated by the PCA method.

**Table 3:** PCA components and the explained variance

Components	Total variance (information)	Cumulative
PCA1	73.62	73.62
PCA2	13.70	87.32
PCA3	4.30	91.62
PCA4	1.82	93.44
PCA5	0.87	94.31
PCA6	0.69	95.00
PCA7	0.62	95.62
PCA8	0.58	96.2

Table 3 shows that the largest variance (73.62%) was provided by the first principal component alone. The second principal component still contained 13.7% of the information. Out of the total of 28 components created, 8 components together contained 96.2% of the information. After the training phase, the predictive performance of the classifiers was tested on the remaining 20% of the data set. To control the accuracy of the ANFIS model in the training phase, the predictive.

Performance of different MFs was tested. In this study, the performance of eight sigmoidal membership functions (psigmf), triangular-shaped (Tri.), trapezoidal-shaped (Trap.), Gaussmf, Gauss2mf, Pimf, Dsigmf, and generalized bell-shaped (Gbell) built-in membership functions were evaluated. The average accuracy of test set data was between 85.88 and 91.57%. The highest values of accuracy, sensitivity, precision, and F-score were 91.57%, 95.18%, 92.31%, and 92.94%, respectively. The results revealed that the ANFIS model with the Gauss2mf built-in MF had the highest accuracy for SA prediction. By selecting the Gauss2mf MF, the accuracy of the SA prediction model reached 92.31%. The results of the comparing MFs are given in Table 4.

**Table 4:** The ANFIS performance based on the different MF types

MFs Type	Precision	Sensitivity	Accuracy	F-score
Gaussmf	0.8621	0.9036	0.8718	0.8824
Gbellmf	0.8851	0.9277	0.8974	0.9059
Trimf	0.8588	0.8795	0.8590	0.8690
Gauss2mf	0.9080	**0.9518**	**0.9231**	**0.9294**
Pimf	**0.9157**	0.9157	0.9103	0.9157
Trapmf	0.8690	0.8795	0.8654	0.8743
Dsigmf	0.8778	**0.9518**	0.9038	0.9133
Psigmf	0.8778	0.9518	0.9038	0.9133

**Fig. 4:**
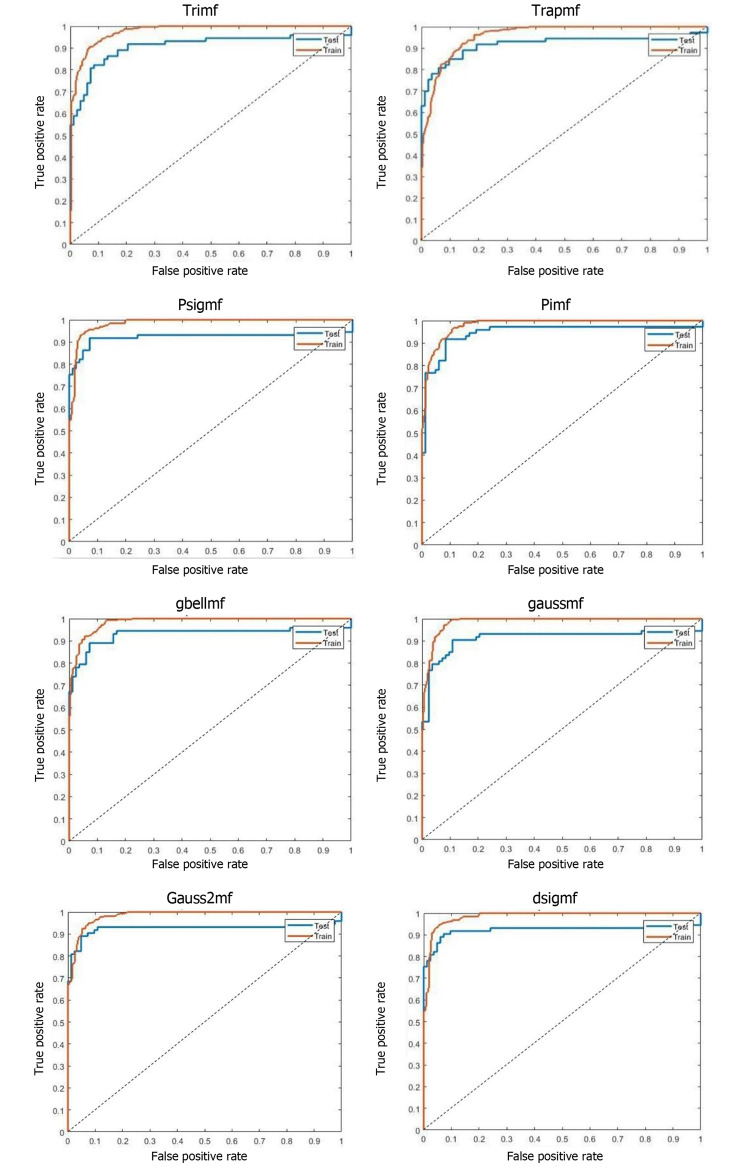
ROC curves according to each MF for training and test sets

The receiver operating characteristic (ROC) diagrams of the ANFIS model based on different MFs are depicted in Fig. 4.

The area under the ROC curve shows the accuracy of the model, which is presented in Fig. 3 for both training and testing sets. If the ROC curve is above the 45-degree line, it is in the desired area, and if the chart is pulled up and to the left, it will be better; the ultimate limit is when it fits perfectly into one. In this diagram, the results were calculated for the desired training and test sets. Table 5 presents the values of the calculated parameters for the ANFIS method according to the types of MFs and other classifiers on the test set.

**Table 5:** Comparison of classifier algorithms for the test set

NO	Methods	TP	FN	FP	TN
1	ANFIS/Gaussmf	**75**	**8**	**12**	**61**
2	ANFIS /Gbellmf	**77**	**6**	**10**	**63**
3	ANFIS /Trimf	**73**	**10**	**12**	**61**
4	ANFIS /Gauss2mf	**79**	**4**	**8**	**65**
5	ANFIS /Pimf	**79**	**4**	**11**	**62**
6	ANFIS /Trapmf	**73**	**10**	**11**	**62**
7	ANFIS /Dsigmf	**79**	**4**	**11**	**62**
8	ANFIS /Psigmf	**79**	**4**	**11**	**62**
9	MLP	**76**	**6**	**12**	**62**
10	SVM	**76**	**9**	**11**	**60**
11	RF	**74**	**12**	**10**	**60**

Table 6 illustrates that the performance of the ANFIS model was superior to the other classifiers in SA prediction.

**Table 6:** Comparison of the performance of the classifiers

ML models	Precision	Sensitivity	Accuracy	F-score
ANFIS (With gauss2MF)	0.9080	0.9518	**0.9231**	0.9294
MLP	0.8636	0.9268	0.8846	0.8941
SVM	0.8735	0.8941	0.8717	0.8837
RF	0.8809	0.8604	0.8589	0.8705

The false-positive rate (FPR) and negative-positive rate (NPR) of the existing methods were analyzed. The FPR measures all non-SA that result in SA based on the classification results. The ANFIS with gauss2MF contains a 10.95% FPR. Still, the MLP, SVM, and RF contain an FPR of 16.21%, 15.49%, and 14.28%, respectively. The false-negative rate (FNR) of the proposed ANFIS method is 4.81%.

In the RF algorithm, for the bag sizes of 100, the performance yielded the best result. Therefore, we considered using a bag size of 100. The next algorithm tested was the SVM. There were 9 trainings performed, and according to the results, a C value of 1 yielded the best performance for all the Kernels used. Moreover, the PUK yielded the best accuracy among the Kernels used. The information on the ANFIS network is presented in Table 7.

**Table 7:** Information on the ANFIS structure

ANFIS structure	Number
Number of nodes	371
Number of linear parameters	180
Number of nonlinear parameters	320
Total number of parameters	500
Number of training data pairs	628
Number of checking data pairs	0
Number of fuzzy rules	20

The structure characteristics of the ANFIS algorithm developed in this research are shown in Table 8:

**Table 8:** Parameter values ​​in the ANFIS algorithm

ANFIS algorithm	Number
Number of repetitions of the training stage	100
The final step of training error	0.1
Initial step	0.01
Step reduction rate	0.8
Step increase rate	1.1

**Fig. 5:**
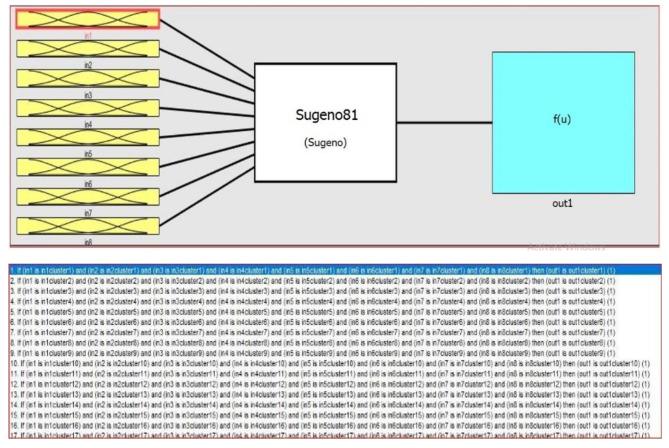
The proposed ANFIS structure

**Fig. 6:**
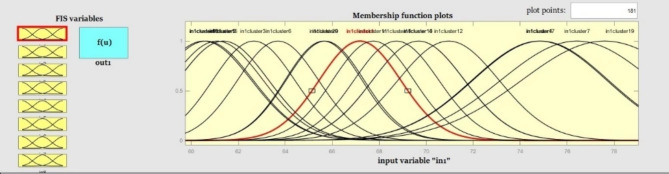
Structures of the membership functions

FCM is a data clustering technique whereby a data set is grouped into m clusters with every instance in the dataset belonging to every cluster to a certain degree. For example, an instance that lies close to the center of a cluster will have a high degree of membership in that cluster, and another data point that lies far away from the center of a cluster will have a low degree of membership in that cluster. FCM clustering is performed by the FCM function. This function uses the mean location of each cluster as a random initial guess for the cluster centers. In the next step, a random membership grade is assigned to each data point using the FCM function for each cluster. The cluster centers and the membership grades for each instance are iteratively updated. After each update, the FCM function moves the cluster centers to the correct location. The iteration goal is to minimize the distance of the data point from a cluster center by an objective function. The membership of that data point in the cluster considers as distance weight. As shown in Figs. 5 and 6, the inputs are eight components of PCA which are finally assigned to the clusters and created the rules. The proposed ANFIS network is presented in Fig. 5.

## Discussion

According to Rowe and Kahn’s theory of SA, this study developed four prediction models based on sociodemographic, cognitive, and functional factors of the elderly to predict SA. Data from developing countries, particularly regarding the elderly, are not common. Such information, in itself, has great importance for reaching an inclusive understanding of the elderly worldwide [71].

After comparing four ML algorithms, the ANFIS model demonstrated the best performance for SA prediction. All these nonlinear ML methods had a satisfactory prediction performance, so for each of them, a minimum precision of 86.36% was obtained. The ANFIS model performed better than the other three models due to its inherent advantages of the fuzzy inference system. The MPL model outperformed RF and SVM. Compared to the MLP model, the ANFIS model has good capabilities due to the integration of the neural network and the fuzzy inference system, including the ability to perform parallel computing, distributed knowledge storage, strong fault tolerance, and the ability of adaptive learning, and it also has a significant capacity to express prior knowledge [62].

So far, several studies have been conducted on the effects of different MFs while developing fuzzy inference systems [72–74]. Herein, we attempted to find what type of MF suits our problem, and among the eight MFs tested, gauss2MF provided the best performance.

In collecting this data set, we gathered the dominant features as much as possible. In the pre-processing stage based on these features, eight components were selected. Several studies have reported important variables for the elderly’s QoL prediction by leveraging feature selection analysis and optimization techniques. The strongest predictive variables in the reviewed studies were categorized into three classes: (1) sociodemographic variables [71, 75–86] such as age, sex, occupation, weight, ethnicity, monthly revenue, and marital status; (2) supportive and lifestyle factors [16, 75, 77, 79, 81, 87–91] such as education level, preventive measures, spirituality status, nutrition factors, physical activity/exercise, residence status, household factors, psychosocial support, domestic violence, living alone, having a partner, family support, number of family member, level of family member caring, accessibility of safe water, social service accessibility, social relationships, insurance situation, justice, and volunteer service; and (3) physical and mental variables [92–94]. such as underlying diseases, number of chronic diseases, hopelessness and depression, discomfort and general malaise, perceived health status, functional status, physical symptoms, and the number of symptoms.

The model proposed in our study accurately predicted the SA of the elderly. Hence, this will likely make the designed model applicable for improving elderly QoL indicators. However, the present study had some limitations that need to be addressed. First, it was a retrospective study in which the existence of some missing and noisy fields (such as incoherent, imbalanced, incomplete, abnormal, meaningless, and erroneous) was inevitable. Therefore, the percentile method was adopted to deal with noisy and outlier fields. Additionally, an eliminate data objects method was applied to handle the missing fields. Second, we used only four ML methods, however, the accuracy and generalizability of our models will be enhanced if we test other ML techniques. Other limitations of the present study were being single-center, the small size of the database, and the lack of external validation for the models’ evaluation which may have adversely affected the generalizability of the data and the quality of modeling. Therefore, further prospective research should be conducted on a data set derived from multiple centers to validate the quality of modeling and minimize prognosis bias. In addition, the external validation method should be used to confirm the results of the present study.

### Suggestions based on the results

Iran is currently in the transition phase of the age structure and is expected to experience the population aging phenomenon in the next few decades. Prevention of disabilities due to old age in this target group can reduce the economic, psychological, and social burden caused by aging. Due to the relatively good accuracy of the proposed model in SA prediction, the development of decision support systems for improving the QoL can play an important role in monitoring and improving the health status of this group. In this regard, embedding the models designed in our study in the form of an intelligent system will help geriatric specialists and senior nurses in providing optimal support services and customized care for the elderly to improve their QoL. Such an intelligent system provides healthcare managers and policymakers with a reliable and responsive tool by predicting adults’ SA based on their sociodemographic, clinical, functional, and mental factors. This predictive model can also be an advantage in increasing SA probability. In future work, this model is expected to be applied and customized to other social problems.

Most data science projects deploy ML models as a demand forecasting service. Some modern applications extend embedded models to mobile devices. On the other hand, web services can provide cheaper and almost instantaneous predictions. If the model runs on a cloud service, the availability of the central processing unit (CPU) power is less of an issue. This model can be easily made available to other applications through an application programming interface (API). The easiest way to deploy an ML model is to create a web service for prediction. Therefore, in our future study, we will deploy the SA prediction model developed in this research as a cloud service available for elderly health monitoring clinics [75, 95, 96].

It is recommended to develop a software for predicting SA based on the proposed model. By using that software in health care centers and in accordance with the predicted result, health policymakers can provide prevention and support programs for SA to prevent future costs.

## Conclusions

The proposed ANFIS-based ML algorithm is an effective model for SA prediction. Using the developed models in real nursing home care environments will improve the elderly’s health-related QoL indicators. In addition, geriatricians and senior nurses can provide personalized and optimized services for the elderly. In future studies, the ANFIS model can be developed by increasing the number of data instances from multi-central datasets.

## Data Availability

The datasets used and/or analyzed during the current study are available from the corresponding author on reasonable request.
